# Quantitative fermentation of unpretreated transgenic poplar by *Caldicellulosiruptor bescii*

**DOI:** 10.1038/s41467-019-11376-6

**Published:** 2019-08-07

**Authors:** Christopher T. Straub, Piyum A. Khatibi, Jack P. Wang, Jonathan M. Conway, Amanda M. Williams-Rhaesa, Ilona M. Peszlen, Vincent L. Chiang, Michael W. W. Adams, Robert M. Kelly

**Affiliations:** 1 Department of Chemical and Biomolecular Engineering, North Carolina State University, Raleigh, NC 27695 USA; 2 Department of Forestry and Environmental Resources, North Carolina State University, Raleigh, NC 27695 USA; 3 Department of Biochemistry and Molecular Biology, University of Georgia, Athens, GA 30602 USA; 4 Department of Forest Biomaterials, North Carolina State University, Raleigh, NC 27695 USA; 5 Present Address: Novozymes Biologicals, Inc., Durham, NC 27709 USA

**Keywords:** Metabolic engineering, Applied microbiology, Bioalcohols

## Abstract

Microbial fermentation of lignocellulosic biomass to produce industrial chemicals is exacerbated by the recalcitrant network of lignin, cellulose and hemicelluloses comprising the plant secondary cell wall. In this study, we show that transgenic poplar (*Populus trichocarpa*) lines can be solubilized without any pretreatment by the extreme thermophile *Caldicellulosiruptor bescii* that has been metabolically engineered to shift its fermentation products away from inhibitory organic acids to ethanol. Carbohydrate solubilization and conversion of unpretreated milled biomass is nearly 90% for two transgenic lines, compared to only 25% for wild-type poplar. Unexpectedly, unpretreated intact poplar stems achieved nearly 70% of the fermentation production observed with milled poplar as the substrate. The nearly quantitative microbial conversion of the carbohydrate content of unpretreated transgenic lignocellulosic biomass bodes well for full utilization of renewable biomass feedstocks.

## Abstract (web summary)

Metabolizing lignocellulosic feedstocks to industrial chemicals by microorganisms requires surmounting  the recalcitrance caused by lignin. Here, the authors pair transgenic lignin modified poplar lines with engineered *Caldicellusiruptor bescii* to achieve biomass solubilization and ethanol conversion without pretreatment.

## Introduction

Industrial biotechnology aspires to produce industrial chemicals from renewable feedstocks to address the finite nature of fossil fuels and associated global warming concerns. Metabolically engineered model microorganisms (typically *Escherichia coli* and yeast) can produce a range of industrial chemicals from simple sugars^1^. However, they are unable to directly metabolize the carbohydrate content of lignocellulosic biomass, such that physical, chemical, and enzymatic pretreatment steps with their associated costs are a bioprocessing necessity. The bacterial genus *Caldicellulosiruptor* is globally distributed in terrestrial hot springs, with some species growing at temperatures up to 90 °C by deconstructing and fermenting the two major complex carbohydrates found in plant biomass: cellulose and hemicelluloses^2^. One species, *Caldicellusiruptor bescii*, can completely solubilize and metabolize crystalline cellulose and hemicellulose in their purified forms^3^. However, lignin, a complex heterogeneous aromatic polymer and the other major component of the secondary plant cell wall, introduces a significant barrier to plant biomass solubilization by this bacterium^4^. Feedstocks with modified lignin content could be the solution to the recalcitrance of lignocellulosic biomass to microbial attack. However, such approaches must align with the physiology of the deconstructing microorganism. Even with switchgrasses transgenically modified to achieve lower lignin levels^5^, *C. bescii* solubilizes at best 36% of the carbohydrate content from unpretreated samples, compared to 24% for the parent wild type; the result for the transgenic is no better than for the solubilization of a natural variant switchgrass by this bacterium^6^. Thus lignin reduction, in and of itself, may not be sufficient to achieve significant microbial conversion of plant biomass. Herein we identify biomass feedstocks with attributes that align with the lignocellulosic features of *C. bescii* so that near-complete carbohydrate solubilization and conversion without pretreatment is achieved.

## Results

### Selection of transgenic poplar lines

Two low lignin, but otherwise unpretreated, transgenic poplar lines^7,8^ were compared with the corresponding parent wild type (22% lignin, syringyl (S)/guaiacyl (G) = 2.1, 4% aldehyde content in lignin) to assess the extent of direct microbial conversion of the carbohydrate content to fermentation products by engineered *C. bescii *(MACB 1058). Line #54 was generated by downregulating the coumarate 3-hydroxylase 3 (*PtrC3H3*) gene (to 12.5% of the wild-type transcript level), resulting in transgenic wood with a lignin content of 10% and a lignin S/G ratio of 9.9. Line #80 targeted the downregulation of two cinnamyl alcohol dehydrogenases (*PtrCAD1* and *PtrCAD2* to 5.9% and 80.9% of the wild-type transcript level, respectively) that reduce cinnamaldehydes to their corresponding alcohols for lignin biosynthesis. This resulted in lowering the lignin content to 14% with an aldehyde content in lignin of 30%. The ultimate goal here was to determine the extent to which direct microbial conversion of the modified lignin poplar lines could be achieved without any physical, chemical, and/or enzymatic pretreatment.

### *C. bescii* conversion of milled transgenic poplar

**Fig. 1:**
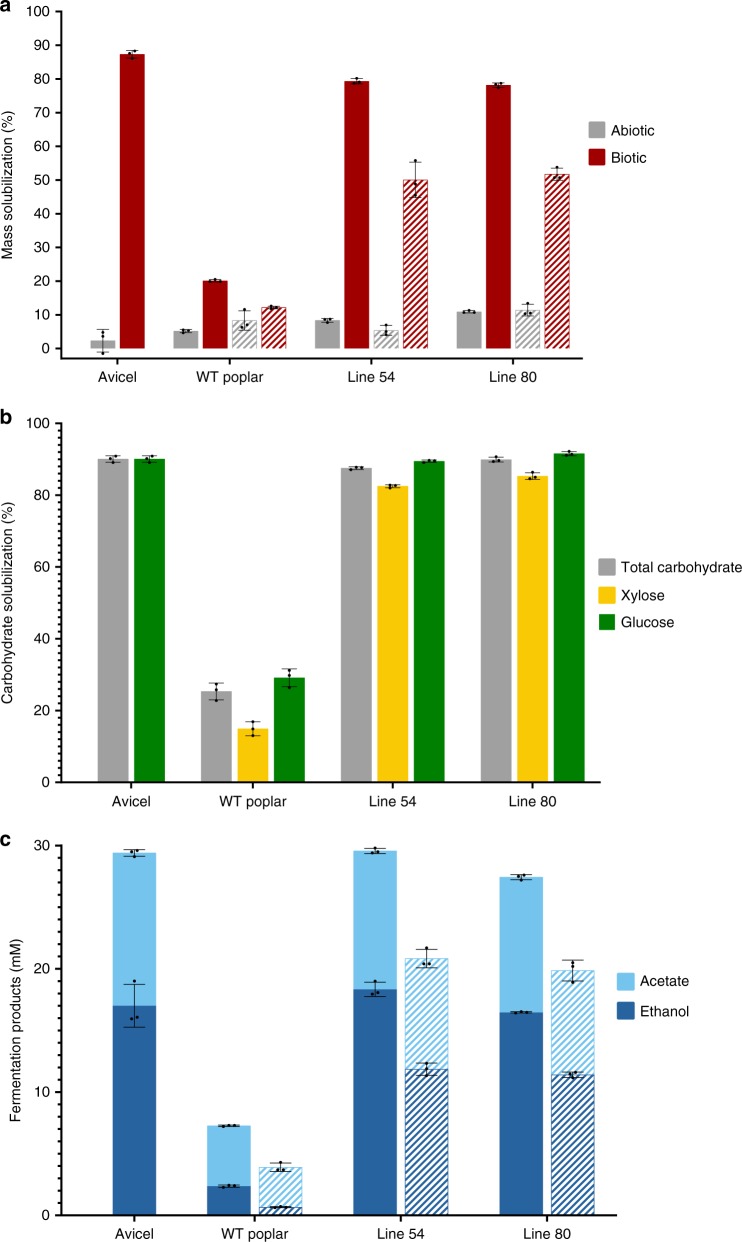
Solubilization and conversion of poplar by engineered *C. bescii*. Greenhouse-grown poplar trees were harvested at 6 months, debarked, air dried, and prepared without further processing as approximately 0.5-cm diameter stem segments. A portion of the stem material was milled and sieved to 40/80 mesh size (0.18-0.42 mm), water washed, dried at 50 °C and used at 5 g/L loading. Further stem segments were cut to a weight of 0.25–0.30 g and used directly in experiments. Solid bars represent milled biomass, hatched bars represent poplar stem segments. **a** Total biomass solubilization. **b** Total carbohydrate solubilization. **c** *C. bescii*  (MACB 1058) fermentation products resulting from 5 g/L substrate loading. Error bars represent one standard error of three biological replicates. Source data are provided as a Source Data file

Based on a previous effort to metabolically engineer *C. bescii*^9^ but using a genetically stable strain of this bacterium^10,11^, the lactate dehydrogenase gene (*ldh*) was deleted to eliminate lactate production, and the bifunctional alcohol dehydrogenase (*adhE)* gene from *Clostridium thermocellum* was inserted to shift fermentation carbon flux to ethanol rather than acetate, reducing product inhibition (*C. bescii* strain *Δldh::adhE*)^12^. The extent of poplar solubilization and conversion by engineered *C. bescii* was quantified in a series of batch fermentations. Cultures were grown on 5 g/L poplar (milled to 0.18–0.42 mm) as the sole carbon source. Total biomass solubilization of the wild-type poplar with engineered *C. bescii* after 7 days at 65 °C (the functional temperature of the AdhE) was 20%, while lines #54 and #80 solubilized 79% and 78%, respectively; abiotic controls in these cases were 5, 8, and 11% for wild type, #54 and #80, respectively (Fig. 1a, Supplementary Tables 1 and 2). Note that purified crystalline cellulose (Avicel) at the same loading was 87% solubilized compared to 2% for the abiotic control. Carbohydrate solubilization for the transgenic lines was nearly complete at 87% and 90% for #54 and #80, respectively, compared to 25% for the wild type; note that carbohydrate release from the transgenic lines was nearly the same as for Avicel (90%), demonstrating the impact of modifying lignin structure and composition (Fig. 1b, Supplementary Table 3). The engineered *C. bescii* strain converted most of the carbohydrate content from the transgenic lines into fermentation products: 18.3 mM/11.3 mM and 16.5 mM/11.0 mM ethanol/acetate for lines #54 and line #80, respectively, compared to 2.4 mM/4.9 mM and 17.0 mM/12.4 mM for wild-type poplar and Avicel, respectively (Fig. 1c, Supplementary Tables 4 and 5).

### *C. bescii* solubilization of transgenic poplar stems

For lignocellulosic biomass bioprocessing, pretreatments and subsequent economic viability concerns typically revolve around heat, chemicals, and enzymes. However, the energy and cost required to reduce the size of the lignocellulose feedstock can likewise have a dramatic impact on the economic considerations. A previous analysis of energy requirements for woody biomass feedstocks demonstrated that chipping mature trees to standard commercial chip size (10–50 mm in two dimensions, 5–15 mm in one dimension) consumes only 0.18 MJ/kg wood, approximately 1% of the total theoretical thermal energy present in the wood. In contrast, chipping followed by disk milling to reduce to fibrous particles (<1 mm) consumes a total of 2.16 MJ/kg wood or >12 times as much energy as chipping alone^13^. Therefore, any commercial bioprocess envisioned, based on extensive size reduction beyond chipping, would face significant economic hurdles.

**Fig. 2:**
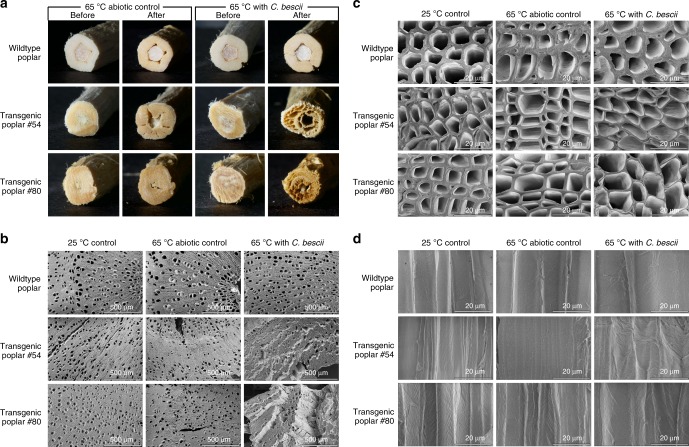
Images of poplar stems with and without solubilization by *C. bescii*. Six-month-old stem segments were incubated for 7 days at 25 °C (25 °C control), at 65 °C without *C. bescii* (abiotic control), or at 65 °C with *C. bescii*. **a** End view of stems before and after treatment indicating the level of degradation. **b** Low-magnification and **c** high-magnification scanning electron microscopic images of stem cross-sections showing splits, fragmentations, and the effects of lignin modification and *C. bescii* solubilization on the xylem cell wall fibers. **d** Comparison of longitudinal xylem fiber cell surfaces for different treatments

Hence, to determine the impact of biomass particle size for the transgenic poplar lines examined here, fermentations utilizing whole segments of 6-month-old trees, approximately 5 mm diameter, were examined. Single segments of the unwashed de-barked stems were cut to obtain a weight of 0.25–0.30 g and subjected to the same conditions as the milled poplar, i.e., 50 mL fermentations with 5–6 g/L loading. At equivalent mass loadings, the stem segments were solubilized at 12% for the wild-type poplar but at 50% for line #54 and 52% for line #80; abiotic controls in these cases were 5, 5, and 11% for wild type, #54 and #80, respectively. Thus the intact transgenic poplar stems, with particle dimensions 100–1000 times larger than the milled material, were solubilized to about two thirds the extent of the comparable milled material. The stems also yielded fermentation products at 70% of that observed with the milled poplar. Surprisingly, measurements of the dried stem segments (diameters and length) following *C. bescii* fermentation revealed that the reduction in length and diameter was minimal. Following biotic treatment, stems were noticeably softer than the abiotic controls, although they retained their shape with no visually obvious degradation. Upon drying, extensive degradation was observed at the ends of the transgenic stems, while the wild-type stems and abiotic controls showed no such degradation (Fig. 2). Bulk density of the wild-type stem decreased by 16% compared to >60% for the transgenic lines, consistent with solubilization and conversion data. *C. bescii* likely accessed carbohydrate content via the cross-sectional vasculature of the plant rather than through radial penetration of the wood. Given that these plant cells range from 5 to 15 μm in diameter and *C. bescii* is a rod-shaped bacterium on order of 1–3 μm in length and 0.5 μm in diameter, this is a plausible hypothesis.

We also investigated the physical changes to the plant cell wall in stem material, brought on by modifying lignin in transgenic poplar, after incubation at 65 °C (abiotic only), and after exposure of the poplar stem at 65 °C to engineered *C. bescii*. Figure 2a shows the cross-section of the stem, demonstrating the dramatic difference following treatment with *C. bescii* at 65 °C. Figure 2b, c show progressively higher-resolution images that reinforce the degradation of the plant vascular structure as carbohydrate is solubilized and converted by *C. bescii*. Note in particular the thinning of the xylem fiber cell wall as a consequence of lignin modification and microbial attack. Figure 2d further illustrates the degradation of the xylem fiber plant cell wall from the longitudinal perspective of the cell wall.

## Discussion

The results here with modified poplar lines can be put into perspective given other recent efforts to solubilize modified lignocellulosic feedstocks. Wild-type *C. bescii* carbohydrate solubilization of unpretreated but milled transgenic switchgrass lines (14.7 g/L biomass loading) at 75 °C was in all cases <30%, and no statistically significant differences were observed when comparing parent and transgenic lines^14^. *C. thermocellum*, a moderately thermophilic anaerobe, was also tested on these feedstocks and performed best on the COMT knock-down (downregulation of the caffeic acid 3-*O*-methyltransferase) switchgrass line with increased carbohydrate solubilization at 60 °C from 45% to 61%. Unpretreated samples of a natural poplar variant with a mutation in a lignin synthesis pathway were also examined with *C. thermocellum* carbohydrate solubilization, but the increase from 20% to 31% for *C. thermocellum* solubilization is far less than the 25–90% carbohydrate solubilization observed here for *C. bescii* acting on lines #54 and #80. Although no carbohydrate conversion data were provided for the *C. thermocellum* poplar and switchgrass solubilization experiments^14,15^, the inability of this bacterium to natively metabolize pentose sugars, which account for 20–50% of the carbohydrate content of lignocellulosic biomass, is clearly a barrier to complete carbohydrate utilization.

A key aspect of the work reported here is that high levels of both solubilization and conversion of plant carbohydrates were achieved without any biomass pretreatment. Not only could *C. bescii* solubilize cellulose and hemicellulose from the transgenic poplar, it also metabolized the resulting pentose and hexose sugars. The results presented here reinforce the prospects for transgenic biomasses as feedstocks for bio-based chemical production by metabolically engineered microorganisms, especially if no pretreatment is required.

## Methods

### Bacterial strains and growth conditions

A previously published recombinant *C. bescii* (MACB 1058) strain was utilized for the solubilization experiments^12^. Briefly, a genetically stable parent strain (MACB 1034—*Δldh*) was utilized with integration of the bifunctional alcohol dehydrogenase (AdhE) from *C. thermocellum* DSM 1313 at the locus between genes Athe_0949 and Athe_0950.

Strains were grown in a shaking incubator oven at 65 °C and 150 RPM in a modified version of DSMZ 671 defined medium under anaerobic conditions, with poplar or Avicel as the sole carbon source.

A freezer stock of the *C. bescii* strain was recovered in media containing 5 g/L cellobiose and then passaged (2% inoculum) onto media containing 0.25 g/L cellobiose and 5 g/L poplar substrate. After 48 h, this was passaged (2% inoculum) onto a medium containing only poplar substrate as the carbon source.

### Poplar substrate preparation

Various lines of genetically engineered *Populus trichocarpa* were generated by Wang et al.^7^. Of the 221 genetically engineered lines analyzed in the aforementioned work, two lines (referred to as #54 and #80) were suggested for further analysis based on previously determined saccharification data and lignin content. For solubilization experiments, the poplar stems from 6-month-old greenhouse-grown trees were stripped of bark and internodes 1–5 were removed. The stems were lightly scraped using a razor blade to remove the outer layers of differentiating xylem cells and then allowed to air dry for 48 h. Small samples of the stems were taken and prepared for further wood chemistry and property examination with data and analysis reported elsewhere^8^. The remainder of the stem samples were cut or ground to size, yet otherwise untreated, for microbial solubilization experiments.

The stem segments were obtained by cutting a section of the stem to a weight of 0.25–0.30 g. The ends of the stems were lightly sanded with 400 grit sandpaper. The stems were dried for 24 h in a 50 °C oven and weighed before utilizing as a substrate in solubilization experiments.

The dry whole stems were milled on a Wiley Mill with a 40-mesh screen and sieved. The 40/80 mesh fraction was collected and washed with 50 °C water by adding 1.5 g of biomass to a 50-mL conical centrifuge tube and filling to approximately 50 mL with deionized (DI) water. The conical centrifuge tube was shaken and then centrifuged for 10 min at 4696 × *g* in a Sorvall X1R swinging bucket centrifuge. The supernatant fraction was carefully removed with a pipette aid. 50 °C DI water was again added to bring the total volume to 50 mL. The conical centrifuge tube was again centrifuged, and the supernatant was removed. This wash was repeated a second time. After the final removal of supernatant, the contents were transferred to an aluminum weigh boat with DI water and allowed to dry at 50 °C for 24 h. The washed and dried material was then utilized in solubilization experiments.

### Poplar substrate solubilization experiments

Serum bottles (150 mL) containing 0.25 g (5 g/L) of poplar substrate (milled, sieved, washed, and dried material or dried stem segments) were filled with 50 mL of DSMZ 671 defined media. Closed bottles were then brought to anaerobic conditions via vacuum purging cycles with 20/80 CO_2_/N_2_. Bottles were inoculated with 2% inoculum and placed in a shaking incubator oven (150 RPM) at 65 °C for 7 days.

Following the microbial fermentation treatment, the stem segment was removed and transferred to a tared weigh boat. The remaining bottle contents, including any residual solids, were transferred to a 50-mL conical centrifuge tube. For milled poplar experiments, all contents were transferred to a 50-mL conical centrifuge tube. The conical centrifuge tubes were centrifuged at 4696 × *g* in a Sorvall Legend X1R centrifuge for 10 min. Supernatant was removed with a pipette aid and sterile filtered for further analysis. Pelleted solids were washed with 50 °C DI water another two times as described above. The biomass pellet was transferred to a tared aluminum weigh boat with DI water. The material was dried at 50 °C for 24 h.

The post-fermentation dry mass includes dry residual solids, dry bacterial weight, and dried stem segment (as applicable). Dry mass (including all solid substrate and dry cell weight) was utilized to calculate mass solubilization and utilized in calculation of carbohydrate balance.

### Calculation of biomass bulk density

Poplar stem mass was measured before solubilization experiments. An average of six diameter measurements was obtained, and along with the stem length, these measurements were utilized to calculate a cylindrical volume as an estimate for bulk volume. Following drying of the stem after biotic treatment, the same procedure was followed to establish a “before and after” estimate of bulk density (mass over calculated cylindrical volume).

### Analysis of fermentation products

Acetate concentrations in the fermentation medium were analyzed by high-performance liquid chromatography (5 mM sulfuric acid mobile phase). A Rezex-ROA column (300 mm × 7.8 mm; Phenomenex) was utilized for separation with detection by a Waters Model 2414 Infrared detector and a Waters Model 2489 Ultraviolet/Visible detector. Ethanol was analyzed via gas chromatography (Shimadzu GC-2014) using an FID detector and Phenomenex ZB-WAXplus column (Part No. 7HK-G013-22).

### Analysis of poplar carbohydrate content

Samples, both pre- and post-fermentation, along with abiotic controls, were analyzed for carbohydrate content via a modified version of NREL procedure—Determination of Structural Carbohydrates and Lignin in Biomass (NREL/TP-510-42618)^6^.

### Electron microscopy

From the treated stem segments, 1-mm-thick disks with duplicates were hand cut using razor blades in wet condition. In addition, longitudinal cuts of approximately 3 mm were also prepared. The disks and the longitudinal sections were attached to stubs and air-dried first, before vacuum dried for examination with FEI Verios 460L field-emission scanning electron microscope.

### Reporting Summary

Further information on research design is available in the Nature Research Reporting Summary linked to this article.

## Supplementary information

Supplementary Information

Reporting Summary

Source Data

## Data Availability

Data supporting the findings of this work are available within the paper and its Supplementary Information files. A reporting summary for this article is available as a Supplementary Information file. The datasets generated and analyzed during the current study are available from the corresponding author upon request. The source data underlying Fig. 1 are provided as a Source Data file.
