# Supplementary material for: Quantitative fermentation of unpretreated transgenic poplar by Caldicellulosiruptor bescii
Source: Nat Commun. 2019 Aug 7;10:3548. doi: 10.1038/s41467-019-11376-6 (PMC6685990; doi:10.1038/s41467-019-11376-6)
Supplement: Supplementary file 1 — Supplementary Information [file 41467_2019_11376_MOESM1_ESM.pdf]

Quantitative fermentation of unpretreated transgenic poplar by *Caldicellulosiruptor bescii*

Straub *et al.*

Supplementary Table 1. Mass solubilization for milled and sieved poplar as well as Avicel.*

	Abiotic (%)	Biotic (%)
**Avicel**	2.30 ± 3.35	87.4 ± 1.15
**Poplar WT**	5.16 ± 0.45	20.1 ± 0.37
**Poplar Line #54**	8.35 ± 0.55	79.3 ± 0.80
**Poplar Line #80**	10.9 ± 0.36	78.2 ± 0.68

*All experiments performed in triplicate (n = 3); mean and standard deviations are reported. Five g/L (dry wt) substrate loading was subjected to 7 day closed bottle microbial (and abiotic control) treatment at 70°C with *C. bescii*.* Data utilized in Fig. 1A.

Supplementary Table 2. Mass solubilization for intact poplar stems.*

	Abiotic (%)	Biotic (%)
**Poplar WT**	8.29 ± 2.87	12.2 ± 0.37
**Poplar Line #54**	5.36 ± 1.47	50.0 ± 5.23
**Poplar Line #80**	11.4 ± 1.76	51.7 ± 1.81

*All experiments performed in triplicate (n = 3); mean and standard deviation are reported. Five g/L (dry wt) substrate loading was subjected to 7 day closed bottle microbial (and abiotic control) treatment at 70°C with *C. bescii*. Data utilized in Fig. 1A.

Supplementary Table 3. Carbohydrate solubilization for milled and sieved poplar as well as Avicel.*

	Glucose Solubilization (%)	Xylose Solubilization (%)	Total Carbohydrate Solubilization (%)
**Avicel**	90.0 ± 0.90	-	90.0 ± 0.90
**Poplar WT**	29.1 ± 2.45	15.0 ± 1.95	25.3 ± 2.31
**Poplar Line #54**	89.5 ± 0.30	82.5 ± 0.46	87.5 ± 0.34
**Poplar Line #80**	91.6 ± 0.59	85.4 ± 0.94	89.9 ± 0.68

*All experiments performed in triplicate (n = 3); mean and standard deviation are reported. Five g/L (dry wt) loading was subjected to 7 day closed bottle microbial treatment at 70°C with *C. bescii*. Data utilized to generate Fig. 1B.

Supplementary Table 4. Fermentation products for milled and sieved poplar as well as Avicel.*

	**Ethanol (mM)**	**Acetate (mM)**
**Avicel**	17.0 ± 1.74	12.4 ± 0.24
**Poplar WT**	2.37 ± 0.09	4.88 ± 0.05
**Poplar Line #54**	18.3 ± 0.57	11.3 ± 0.21
**Poplar Line #80**	16.5 ± 0.05	11.0 ± 0.22

*All experiments performed in triplicate (n = 3); mean and standard deviation are reported. Five g/L (dry wt) substrate loading was subjected to 7 day closed bottle microbial treatment at 70°C with *C. bescii*. Data utilized in Fig. 1C.

Supplementary Table 5. Fermentation products for poplar stems.*

	**Ethanol (mM)**	**Acetate (mM)**
**Poplar WT**	0.66 ± 0.05	3.23 ± 0.31
**Poplar Line #54**	11.9 ± 0.50	8.98 ± 0.77
**Poplar Line #80**	11.4 ± 0.23	8.47 ± 0.88

*All experiments performed in triplicate (n = 3); mean and standard deviation are reported. Five g/L (dry wt) substrate loading was subjected to 7 day closed bottle microbial treatment at 70°C with *C. bescii*. Data utilized in Fig. 1C.
